# 3D Printing of Bone Grafts for Cleft Alveolar Osteoplasty – *In vivo* Evaluation in a Preclinical Model

**DOI:** 10.3389/fbioe.2020.00217

**Published:** 2020-03-25

**Authors:** Paula Korn, Tilman Ahlfeld, Franziska Lahmeyer, David Kilian, Philipp Sembdner, Ralph Stelzer, Winnie Pradel, Adrian Franke, Martina Rauner, Ursula Range, Bernd Stadlinger, Anja Lode, Günter Lauer, Michael Gelinsky

**Affiliations:** ^1^Department of Oral and Maxillofacial Surgery, Charité – Universitätsmedizin Berlin, Corporate Member of Freie Universität Berlin, Humboldt-Universität zu Berlin, and Berlin Institute of Health, Berlin, Germany; ^2^Centre for Translational Bone, Joint and Soft Tissue Research, University Hospital “Carl Gustav Carus”, Faculty of Medicine, Technische Universität Dresden, Dresden, Germany; ^3^Department of Oral and Maxillofacial Surgery, Faculty of Medicine “Carl Gustav Carus”, Technische Universität Dresden, Dresden, Germany; ^4^Institute of Machine Elements and Machine Design, Technische Universität Dresden, Dresden, Germany; ^5^Division of Endocrinology, Diabetes, and Bone Diseases, Department of Medicine III and Center for Healthy Aging, Faculty of Medicine “Carl Gustav Carus”, Technische Universität Dresden, Dresden, Germany; ^6^Institute for Medical Informatics and Biometry, Faculty of Medicine “Carl Gustav Carus”, Technische Universität Dresden, Dresden, Germany; ^7^Clinic of Cranio-Maxillofacial and Oral Surgery, University Hospital Zurich, University of Zurich, Zurich, Switzerland

**Keywords:** 3D printing, bone graft, bone tissue engineering, alveolar cleft model, calcium phosphate cement

## Abstract

One of the most common hereditary craniofacial anomalies in humans are cleft lip and cleft alveolar bone with or without cleft palate. Current clinical practice, the augmentation of the persisting alveolar bone defect by using autologous bone grafts, has considerable disadvantages motivating to an intensive search for alternatives. We developed a novel therapy concept based on 3D printing of biodegradable calcium phosphate-based materials and integration of osteogenic cells allowing fabrication of patient-specific, tissue-engineered bone grafts. Objective of the present study was the *in vivo* evaluation of implants in a rat alveolar cleft model. Scaffolds were designed according to the defect’s geometry with two different pore designs (60° and 30° rotated layer orientation) and produced by extrusion-based 3D plotting of a pasty calcium phosphate cement. The scaffolds filled into the artificial bone defect in the palate of adult Lewis rats, showing a good support. Half of the scaffolds were colonized with rat mesenchymal stromal cells (rMSC) prior to implantation. After 6 and 12 weeks, remaining defect width and bone formation were quantified histologically and by microCT. The results revealed excellent osteoconductive properties of the scaffolds, a significant influence of the pore geometry (60° > 30°), but no enhanced defect healing by pre-colonization with rMSC.

## Introduction

During embryologic development of the craniomaxillofacial anatomy, tissue fusion is essential. In cases of non- or incomplete fusion, soft and/or hard tissue defects will remain after birth. The most common examples for congenital craniofacial anomalies caused by an incomplete tissue fusion are cleft lip and cleft alveolus with or without a cleft palate. The prevalence in humans varies between different ethnical groups and averaged for Caucasians at one per 700 live births (Dixon et al., 2011). Three of four cleft lip and palate patients expose an alveolar osseous defect (Guo et al., 2011). Children suffering from a complete cleft palate, alveolus and lip require an extensive treatment from their first months until adolescence. One aspect of the surgical therapy is the augmentation of the persisting alveolar bone defect, called alveolar cleft osteoplasty, by autologous bone grafts. This has to be performed at the age of 9–11 years in order to create sufficient bone volume, allowing the eruption of the permanent canine. Additionally, bone grafting of this defect normalizes facial and dental function. Failure to reconstruct this osseous deformity may result in oronasal fistula, fluid reflux, speech pathology, anteroposterior deficiency of the maxilla, transverse deficiency of the maxilla, lack of bone support for the teeth, dental crowding, and facial asymmetry (Waite and Waite, 1996).

Up to now, the alveolar cleft osteoplasty uses mainly autologous bone grafts from the iliac crest, providing osteogenic, osteoinductive and osteoconductive properties (Lopez et al., 2018). A disadvantage of these bone grafts is the need of harvesting with associated donor site morbidity. In cases of maxillofacial and cleft reconstructions, the following rates of complications in the donor site region are summarized by a review of Boehm et al. (2018): acute (45.7%) and chronic (1.5%) gait disturbance, acute (17.8%) and chronic nerve changes (1.4%), hypertrophic/painful scar (9.1%), chronic pain (3.1%), hematoma (2.2%), seroma (2.0%), infection (1.0%) and iliac crest fracture (1.2%). Against this background, it is of great clinical interest to evaluate biomaterials regarding their potential to be an alternative for the autologous bone grafts. For small oral bone defects alloplastic and xenogenic materials are well established, but for congenital and critical size defects the clinical results of these materials are currently not sufficient (Feinberg et al., 1989). Hence, there are great efforts to develop bone grafts, which can be used for the reconstruction of critical size defects like alveolar clefts (Silva Gomes Ferreira et al., 2018). The bone grafts should have the following characteristics: osteoconductivity, mechanical stability, promotion of vascular ingrowth and stem cell recruitment, and progressive resorption during the replacement by native tissue (Hutmacher et al., 2007; Kim et al., 2017).

Ideally, the bone graft is patient-specific and fits exactly into the defect area. This can be achieved by additive manufacturing of an implant based on a 3D model that is designed by using computer tomography (CT) or magnet resonance tomography (MRT) data of the defect region (Pfister et al., 2004). In the last two decades, various methods of additive manufacturing have been adapted to the fabrication of medical implants and tissue engineering constructs, amongst them extrusion-based fabrication methods (Landers et al., 2002; Malda et al., 2013). Extrusion printing in mild conditions, for example by avoidance of unphysiological temperatures, pH or energy-intensive post-printing treatment, is called 3D plotting (Moroni et al., 2018). The mild processing conditions allow the combined processing of materials and biological substances like proteins/growth factors and cells (called bioplotting) (Fedorovich et al., 2008; Poldervaart et al., 2013).

The regeneration of tissue defects is accomplished by cells; the biomaterial just fills the defect volume and provides a supporting substrate by mimicking the extracellular matrix. The cells invade into the defect region from the surrounding tissue or the biomaterial scaffold is pre-colonized with regenerative cells according to the tissue engineering concept; growth factors and other signaling molecules can be integrated in the material to stimulate cellular reactions necessary for tissue regeneration such as migration, proliferation and differentiation (Hutmacher et al., 2007). Previous preclinical studies investigated tissue engineering approaches in the context of alveolar cleft osteoplasty by testing calcium phosphate-based biomaterials which were pre-colonized with rat mesenchymal stromal cells (rMSC) (Korn et al., 2014, 2017) or coated with bone morphogenetic protein 2 (BMP-2) (Nguyen et al., 2009) in rat alveolar cleft models. The mentioned material combinations showed local bone formation within the artificial defect, but no complete osseous defect healing. First trials of clinical application of tissue engineered bone grafts led to promising results. An example is the study of Pradel and Lauer (2012), who found comparable rates of bone formation for pure autologous (from iliac crest) and mixed alloplastic (autologous osteoblasts obtained from maxilla bone biopsies and cultured on demineralized bone matrix Osteovit bone grafts). Guo et al. (2011) reported for collagen bone graft substitutes impregnated with BMP-2 (InFuse bone graft, Sofamor-Danek, United States) comparable results. However, most studies have in common, that the small number of patients might led to a high bias of the results and up to now no real alternative bone graft is clinically established (Liang et al., 2018).

We aim to develop a novel concept for the treatment of alveolar cleft patients by the combination of 3D plotting of patient-specific bone implants and tissue engineering approaches; due to their resemblance of the natural bone mineral, we focus on calcium phosphate-based materials. Herein, we utilized a plottable, clinically approved calcium phosphate cement paste (CPC), which is composed of calcium phosphate precursors (mainly α-tricalcium phosphate) and a biocompatible, but hydrophobic (oil-based) carrier liquid (Heinemann et al., 2013; Lode et al., 2018). This composition allows long storage and unlimited extrusion in mild conditions (Lode et al., 2014). Macroporous CPC scaffolds can be plotted with high accuracy, afterward the scaffolds need to be hardened in an aqueous environment (Akkineni et al., 2015; Ahlfeld et al., 2017). During this setting procedure, the carrier liquid vanishes from the scaffold structure without residues and the precursors set to nanocrystalline calcium-deficient, carbonated hydroxyapatite, which can be resorbed by osteoclasts (Bernhardt et al., 2014; Reitmaier et al., 2018). The mechanical properties of bulk and plotted CPC samples were characterized thoroughly in the past (Ahlfeld et al., 2017; Lode et al., 2018). Patient-specific scaffold structures can be fabricated by 3D plotting of CPC (Ahlfeld et al., 2018b).

In the present study, we analyzed custom-made bone grafts consisting of 3D plotted CPC scaffolds and rMSC in a small animal model of cleft alveolar osteoplasty. The hypothesis of the study was “The application of a 3D plotted bone graft into an artificial maxillary bone defect leads to a significant reduction of the defect width after 12 weeks.” To better understand the performance of 3D plotted implants, we investigated the influence of the fabricated pore geometry on bone formation in the defect area, as well as the effect of rMSC seeded onto the scaffold prior to implantation.

## Materials and Methods

### 3D Plotting of CPC Scaffolds

The plottable CPC paste (INNOTERE Paste-CPC), manufactured by INNOTERE GmbH (Radebeul, Germany), was sterilized by γ-irradiation (25 kGy) and transferred into cartridges (Nordson EFD, Oberhaching, Germany) which were placed into a three-axis robotic dispensing system (Bioscaffolder 3.1, GeSiM mbH, Radeberg, Germany). For the *in vitro* and *in vivo* study, scaffolds with a diameter of 3.0 and 3.2 mm and a height of 0.48 mm (four layers) were plotted utilizing a 230 μm needle (Globaco GmbH, Rödermark, Germany) with a plotting speed of 10 mm⋅s^–1^ and an air pressure of 150 kPa. Inner geometry of the scaffolds was adjusted as follows: strand-to-strand distance 0.5 mm, layer-to-layer orientation 60° (Scaffold A) or 30° (Scaffold B). After plotting, scaffolds were incubated for 3 days in water-saturated atmosphere (humidity > 95%, temperature 37.4°C) (Akkineni et al., 2015), followed by three intensive washing steps in acetone to remove residual oil of the CPC paste. The whole fabrication process was conducted under sterile conditions. Scaffolds were immersed in cell culture medium consisting of alpha-MEM (Gibco, Thermo Fisher Scientific GmbH, Germany) with 10% fetal calf serum (FCS), 100 Uml^–1^ penicillin and 100 mgml^–1^ streptomycin (Pen/Strep, all from Biochrom, Berlin, Germany) 24 h prior to subsequent cell seeding. The anatomical model was virtually segmented, modified and constructed with the softwares Dornheim segmenter (Dornheim Medical Images GmbH, Magdeburg, Germany), Geomagic Studio (RSI 3D-Systems, Oberursel, Germany) and Geomagic Freeform (RSI 3D-Systems, Oberursel, Germany). As sacrificial ink a 10% methylcellulose (mc, M0512, Sigma, United States, molecular weight ≈88000 Da, 4000 cP) paste was prepared in water as described before (Ahlfeld et al., 2018b). The sacrificial ink was plotted utilizing a 410 μm needle with a speed of 10 mm⋅s^–1^; after post-processing, it was washed away in the fridge overnight.

### Seeding of the Scaffolds With rMSC for *in vitro* and *in vivo* Experiments

rMSC were isolated from the bone marrow of adult Lewis rats as described previously (Korn et al., 2017). In brief, bone marrow was aspirated from the femur, centrifuged for 10 min at 1200 rpm and the pellet was resuspended in cell culture medium consisting of alpha-MEM with 10% fetal calf serum, Pen/Strep, 1% Amphotericin and 1 M HEPES buffer solution (all from Gibco, Thermo Fisher). The cell suspension was transferred into culture flasks; the medium was changed every 3–4 days and the cells were expanded until the second passage. For cell seeding, the immersed scaffolds were placed into 0.2 ml PP multiply-pro cups (Sarstedt; one scaffold per tube) and 280 μl cell suspension containing either 1 × 10^5^ cells (for the *in vitro* experiments) or 2 × 10^5^ cells (for the *in vivo* experiments) were added. The scaffold colonization was performed by a rotation method: during a period of 6 h, the tubes were rotated every 30 min one and a half turn while they were stored in the incubator at 37°C and 5% CO_2_. Finally, the scaffolds were placed in 96-well plates which were filled with cell culture medium. For the *in vivo* experiments, the bone grafts stayed in the incubator at 37°C, 5% CO_2_ and 95% humidity for 3 days until implantation. The scaffolds which were not colonized with cells were treated with the same procedure to ensure same conditions for the material prior to implantation.

### *In vitro* Experiment

Seeded scaffolds were cultivated in cell culture medium at 37°C, 5% CO_2_ and 95% humidity for 28 days; for half of the samples osteogenic supplements (10^–7^ M dexamethasone, 0.05 mM ascorbic acid 2-phosphate, 10 mM beta-glycerophosphate; all from Sigma-Aldrich) were added to the medium starting 1 day after seeding. For fluorescence microscopic analyses, cell-colonized scaffolds were fixed using 4% formaldehyde and actin cytoskeletons and cell nuclei were stained with AlexaFluor 488^®^ phalloidin (Invitrogen) and DAPI (Sigma-Aldrich); imaging was performed with a Keyence BZ9000E. For biochemical analysis of LDH and ALP activity, the samples, frozen at different time points of cell culture, were thawed and incubated with lysis buffer (1% Triton X-100 in PBS) for 50 min on ice; cell lysis was supported by sonication for 10 min. LDH activity in the lysates was determined with the CytoTox 96 Non-radioactive Cytotoxicity Assay (Promega) according to the manufacturer’s instruction and correlated with the cell number using a calibration line. Measurement of ALP activity was done as described previously (Lode et al., 2014). In brief, an aliquot of the lysate was incubated with 1 mg ml^–1^*p*-nitrophenylphosphate (Sigma-Aldrich) in ALP substrate buffer (0.1 M diethanolamine, 1% Triton X-100, 1 mM MgCl_2_, pH 9.8) at 37°C for 30 min. The enzymatic reaction was stopped by addition of 1 M NaOH and the *p*-nitrophenolate (pNp) formation was quantified by measurement of the absorbance at 405 nm. Using a *p*-nitrophenol calibration line, the amount of pNp produced by the cell lysate was calculated and related to the cell number in each sample (calculated from the LDH activity).

### *In vivo* Application in a Rat Alveolar Cleft Model

The animal study was approved by the Commission for Animal Studies at the District Government Dresden, Germany (DD24-5131/354/26). For the study, 80 adult male Lewis rats (Janvier Labs, Le Genest-Saint-Isle, France) with an average body weight of 450 g and an age of 6 months at the beginning were used. All animals were housed according to the current regulations in a light- and temperature-controlled environment. They had access to water *ad libitum* and were fed with pellets (ssniff-Spezialdiäten GmbH, Soest, Germany). After statistical calculation of the required number of animals per group all rats were randomly divided into the 5 experimental groups (see Table 1). The rats were anesthetized by intraperitoneal injection of ketamine (100 mg/kg body weight) and xylazine (10 mg/kg body weight) and fixed in a dorsal position. An artificial alveolar cleft was created surgically in the anterior maxilla of each animal. First, a sagittal incision was made following the mid-palatal suture. After elevation of a mucosal flap and removal of the periosteum, a localized bone defect with 3.3 mm in diameter was created using a diamond-coated cylindrical shaped drill (DiT Dental-Instrumente GmbH, Oberlungwitz, Germany). According to the randomized distribution, each rat received one bone graft (Table 1). After insertion of the bone graft, the flap was repositioned and wound closure was performed using 5-0 Ethilon suture (Ethicon, Norderstedt, Germany). Postoperatively, the animals received amoxicillin trihydrate (Fort Dodge Veterinär GmbH, Würselen, Germany) 15 mg/kg body weight once and 4 mg/kg body weight carprofen (Rimadyl;Pfizer Deutschland GmbH) every 24 h for 4 days. All drugs were injected subcutaneously. The animals were fed with a soft diet for the first 3 days and, subsequently, received a regular diet. Postoperatively, the animals and their behavior were monitored and the body weight was measured every 2 weeks. For the *ex vivo* assessment of the dynamic bone formation, all rats received intraperitoneal injections of the fluorochrome dyes alizarine (20 mg/kg body weight) and calcein (30 mg/kg body weight) 7 and 3 days prior to sacrifice.

**TABLE 1:** Experimental groups investigated *in vivo*.

Number of animals	Bone graft	Description	Healing time [weeks]
8	Scaffold A	60° layer rotation	6
8	Scaffold A		12
8	Scaffold A + rMSC		6
8	Scaffold A + rMSC		12
8	Scaffold B	30° layer rotation	6
8	Scaffold B		12
8	Scaffold B + rMSC		6
8	Scaffold B + rMSC		12
8	Control	Empty defect	6
8	Control		12

### Evaluation Methods

After sacrifice, the cranium of each rat was dissected and fixed in 4% formaldehyde. MicroCT and preparation of the histological samples followed.

#### MicroCT

One 2D-microCT per rat was performed *ex vivo* using a VivaCT (SCANCO Medical AG, Brüttisellen, Switzerland) with the following adjustments: x-ray energy 70kVp and 114 mA, integration time 200 ms, voxel size 30 μm and conebeam continuous rotation. A 3D reconstruction of the defect area by Software Script (SCANCO) followed. The fitting accuracy of the scaffolds were characterized descriptively.

#### Histology

After dehydration in a graded series of ethanol, all samples were embedded in methylmethacrylate (Technovit 9100, HeraeusKulzer, Wehrheim, Germany) as described previously (Korn et al., 2014). Coronal sections were produced according to Donath’s sawing and grinding technique (Donath and Breuner, 1982). Thus, the four central sections of each specimen could be achieved for evaluation. Subsequently, the sections measuring 60 μm in thickness were polished. After analysis of the fluorochrome marker uptake, Masson-Goldner trichrome staining followed.

#### Histological Analysis

All samples were imaged by fluorescence microscopy and, after staining, by light microscopy (Olympus BX 61, Olympus Deutschland GmbH, Hamburg, Germany) using cell^F Imaging Software for Life Science (Olympus). Multiple image alignment was performed using an automatic scanning table (Märzhäuser, Wetzlar, Germany). Thus, 8 images per sample were scanned with a 10 × 10-fold magnification and manually fused to one image. Fluorochrome marker uptake was analyzed to assess the dynamics of bone formation at the defect margins. Focus were the direction and distribution of the bone formation marked by the red fluorescent alizarin and the green fluorescent calcein. Thereafter, all specimens were stained according to Masson-Goldner trichrome staining. A descriptive analysis evaluated the position of the scaffold, it’s surface, the interactions between host bone and bone graft and the bone formation on the defect margins. Again, a 10 × 10-fold magnification was chosen. For quantification of the osseous healing the following parameter were measured: remaining defect width (Equation 1), bone formation in the defect area, and percentage of the new formed bone relating to the particular initial defect area (Equation 2). All measurements were realized by one examiner who was masked regarding to the experimental groups.

(1) $$r\,e\,m\,a\,i\,n\,i\,n\,g\,d\,e\,f\,e\,c\,t\,w\,i\,d\,t\,h=\frac{d\,i\,s\,t\,a\,n\,c\,e_{d\,e\,f\,e\,c\,t\,m\,a\,r\,g\,i\,n\,s\,c\,r\,a\,n\,i\,a\,l}+d\,i\,s\,t\,a\,n\,c\,e_{d\,e\,f\,e\,c\,t\,m\,a\,r\,g\,i\,n\,s\,c\,a\,u\,d\,a\,l}}{2}$$

(2) $$n\,e\,w\,l\,y\,f\,o\,r\,m\,e\,d\,b\,o\,n\,e=\frac{n\,e\,w\,f\,o\,r\,m\,e\,d\,b\,o\,n\,e\,a\,r\,e\,a}{i\,n\,i\,t\,i\,a\,l\,o\,s\,s\,e\,o\,u\,s\,d\,e\,f\,e\,c\,t\,a\,r\,e\,a}\times 100$$

#### Statistics

Results obtained *in vitro* were checked for statistical significance by one-way ANOVA coupled with Tukey’s multiple comparison test utilizing GraphPad Prism version 8 software (GraphPad Software, La Jolla, CA, United States).

Statistical analysis of the *in vivo* results were performed with SPSS 25 software (IBM Germany, Ehningen, Germany) and mean as well standard deviations were calculated for all groups. The impact of scaffold and healing time were tested by a two-way-ANOVA. The interactions between healing time, scaffold and rMSC colonization could be studied by *t*-tests with Bonferroni-adjustment. For all analysis the level of significance was set at 95% (*p* = 0.05).

## Results

### Tissue Engineered, Bioresorbable Bone Grafts for Artificial Cleft Palates in Lewis Rats

#### Tissue Engineering Concept

**FIGURE 1:**
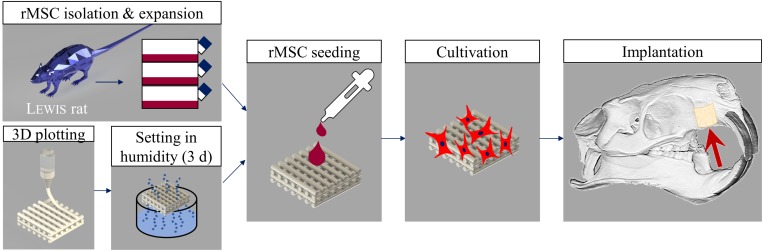
Tissue engineering approach carried out in this study. Rat MSC were isolated from bone marrow of Lewis rats, expanded and seeded onto 3D plotted CPC scaffolds. After 3 days of cultivation, the tissue engineered bone grafts were implanted. Cell-free scaffolds were used as control.

**FIGURE 2:**
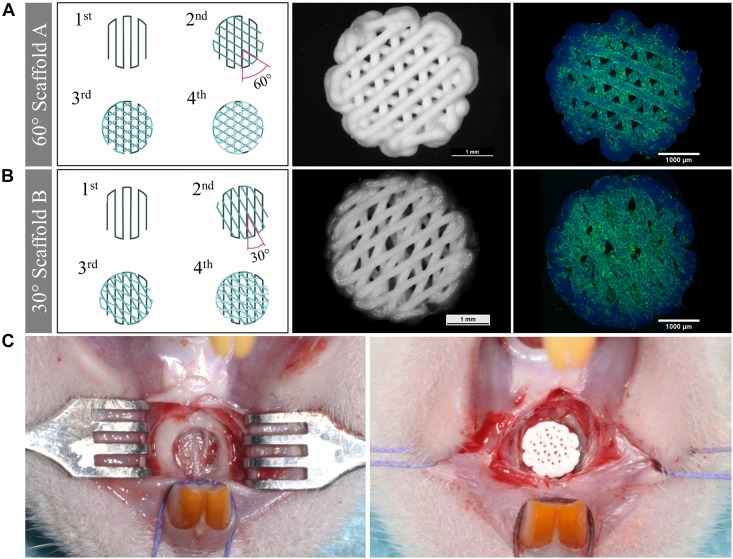
Morphology of scaffolds of type A and B, cell colonization and implantation. **(A,B)** Scheme of the scaffolds, stereomicroscopical images of the scaffolds after plotting and fluorescence-microscopical images of the scaffolds seeded with rMSC (green: actin cytoskeletons, blue: nuclei, CPC exhibits blue autofluorescence). Scaffolds of type A were fabricated with 60° layer-to-layer orientation and scaffolds of type B with 30° layer-to-layer orientation. **(C)** Empty defect of the cleft (left) and filled defect with a precisely fitting scaffold (right).

The tissue engineering concept investigated in this study is shown in Figure 1. Allogenic mesenchymal stromal cells (rMSC) were isolated from bone marrow of Lewis rats and expanded until passage 2. The transfer of allogenic cells causes no immunological problems, as Lewis rats are an inbreeding breed of genetically identical animals. Nevertheless, during the later clinical application, we favored the usage of autologous cells. Miniaturized, precisely fitting scaffolds consisting of CPC were plotted in 60° (scaffold A) and 30° (scaffold B) layer-to-layer orientation and post-processed by setting in water-saturated atmosphere (Figures 1, 2A,B). After setting, the strand widths of the scaffolds A and B were determined as 199.8 ± 9 μm and 195.6 ± 9 μm, respectively. Next, scaffolds were colonized with the isolated rMSC according to the classical tissue engineering approach; actin/nuclei stainings of the scaffolds A and B seeded with rMSC are shown in Figures 2A,B. After a cultivation time of 3 days, scaffolds were implanted into the Lewis rats. To investigate the influence of rMSC on healing, cell-free samples were used as controls. All scaffold types, no matter on layer orientation or cell population, fitted precisely into the artificial alveolar cleft (Figure 2C).

#### *In vitro* Evaluation

**FIGURE 3:**
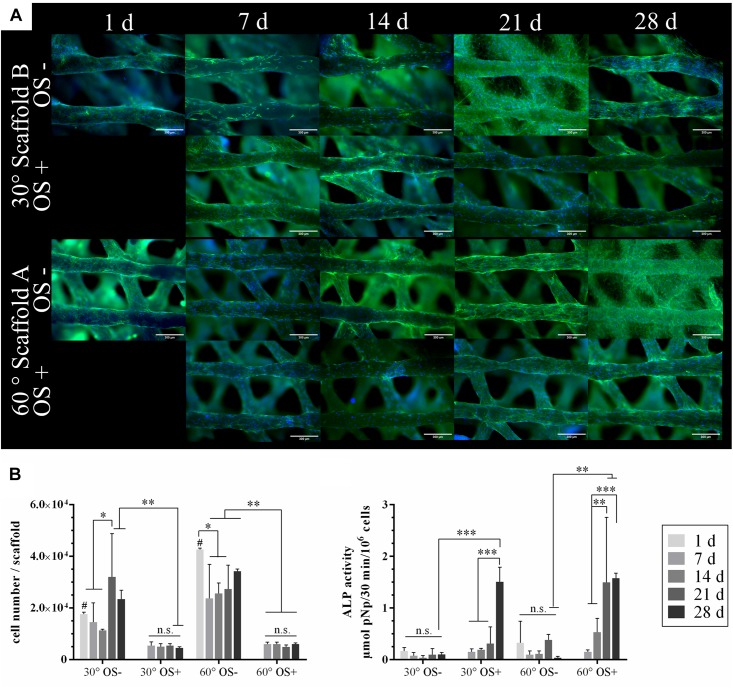
Colonization of the plotted CPC scaffolds with rMSC and osteogenic differentiation *in vitro*. **(A)** Stainings of actin cytoskeletons (green) and nuclei (blue) of rMSC on the scaffold types A and B. CPC revealed a blue autofluorescence. Scale bars represent 300 μm. **(B)** Cell number analyzed by LDH activity and specific ALP activity of the rMSC cultured with (OS+) and w/o (OS–) osteogenic supplements (*n* = 4, mean ± SD, ^∗^*p* < 0.05, ^∗∗^*p* < 0.01, ^∗∗∗^*p* < 0.001, # marks the difference between 30° and 60° with *p* < 0.05, n.s., no significance).

Scaffolds of type A and B were seeded with 1 × 10^5^ rMSC and cultivated over 28 days in cell culture medium with and without osteogenic supplements (OS), respectively. At various time points, cell distribution and density on the scaffolds were visualized by fluorescence microscopy after staining cell nuclei and actin cytoskeletons (Figure 3A); the number of cells grown on the scaffolds was determined by measurement of lactate dehydrogenase (LDH) activity and osteogenic differentiation was evaluated by measurement of alkaline phosphatase (ALP) activity (Figure 3B).

Both, the microscopic analysis and the analysis of LDH activity as a measure for the number of viable cells indicated a significant higher initial cell number (day 1) for scaffold A compared to scaffold B. However, in both cases, a uniform cell distribution on the scaffolds was achieved by the rotation seeding method (see Materials and Methods). The seeding efficiency was approx. 20% in case of scaffold B and nearly 45% in case of scaffold A. After day 1, the number of cells decreased first but increased after day 7 when cultured without osteogenic supplements (OS−); in the presence of osteogenic supplements (OS+), the decrease of cell number was even stronger, staying constant during further cultivation (Figure 3B). Nevertheless, microscopic analyses revealed a complete and uniform coverage of the CPC strands in all cases (Figure 3A). An increase of the ALP activity was detected for cells cultivated with osteogenic supplements (OS+), but not without (OS−), indicating their differentiation toward the osteoblastic lineage after stimulation; no significant effect of the pore geometry (scaffold A vs. B) has been observed (Figure 3B).

### *In vivo* Evaluation

#### Design of the *in vivo* Study and Post-operative Evaluation

Table 1 lists all groups investigated in this study. Scaffolds of type A and B were implanted either with or w/o cells (seeding cell number 2 × 10^5^). Healing times were either 6 or 12 weeks. An empty defect was chosen as control. The study was completed by 78 of 80 rats, which represents a survival rate of 97.5%. Two rats died 3 days after surgery due to unknown reason. They were replaced and finally in all groups 8 rats has been analyzed. Unfortunately, 2 bone defects of the group Scaffold B + rMSC after 6 weeks of healing time were prepared too flat, as visible in the histological sections, and hence were excluded from the evaluation 74 of 80 scaffolds did not show signs of ruptures due to mechanical load caused by chewing. Five of the six fractured scaffolds belong to the group B with the 30° strand rotation. Nevertheless the fractured fragments were not dislocated into the area outside of the defect. The rats’ body weight at the end of the study was comparable to the initial weight and all rats showed an undisturbed behavior. No clinical wound healing complications were observed.

#### Microcomputed Tomography Analysis

**FIGURE 4:**
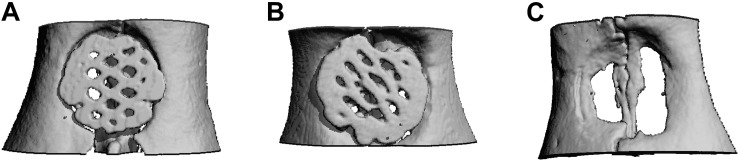
Micro-CT after 12 weeks. Exemplary axial sections are shown for **(A)** Scaffold A, **(B)** Scaffold B, and **(C)** control group.

In μCT images, lamellar and cancellous bone as well as the scaffolds were isodense. Nevertheless discrimination between bone and scaffold was possible due to morphological characteristics. Rarely a fusion of bone and scaffold margin was visible. The descriptive analysis of the scaffold position, being performed in axial and coronal sections, showed no differences between the groups (Figure 4). In some cases, the scaffold was inserted too far in cranial or caudal direction. Further an angular scaffold position occurred as well as six scaffolds (5x scaffold B, 1x scaffold A) were fractured at the measurement time point.

#### Histomorphology: Polyfluorochrome Labeling

**FIGURE 5:**
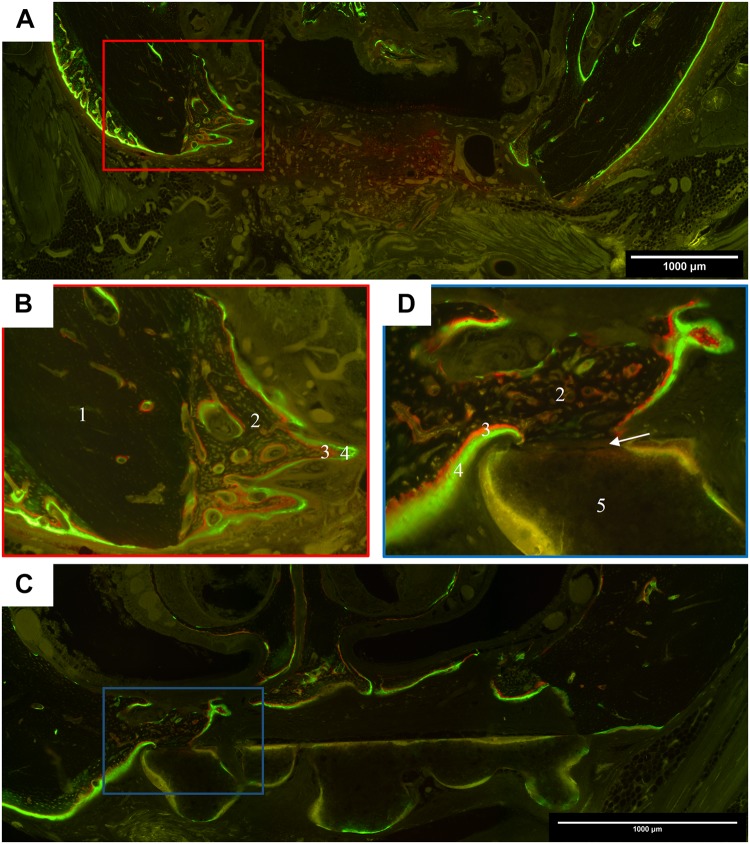
Representative images of polyfluorchrome labeling with alizarin and calcein in coronal histological sections. **(A)** Control group after 6 weeks healing time; 10 × 10 magnification; cone-like shaped bone formation started from the former defect margins, which led to a partial ossification of the defect area, but no complete osseous healing. **(B)** Detail of the defect margin of image A, 20 × 10 magnification; green calcein labels were followed by red alizarin labels, showing that cancellous bone grew from the former defect margin toward the center. **(C)** Scaffold A group after 6 weeks healing time; 10 × 10 magnification; the scaffold is located in the defect center and cancellous bone grew on the cranial site of its surface. **(D)** Detail of the defect margin of image C, 20 × 10 magnification, newly formed bone with dense contact to the scaffold. In the contact zone, the labeling is interrupted. (*1 lamellar host bone, 2 new formed cancellous bone, 3 alizarin label, 4 calcein label, 5 scaffold, arrow represents the contact zone between scaffold and bone*).

For the *ex vivo* assessment of the dynamic bone formation, all rats received intraperitoneal injections of the fluorochrome dyes alizarine and calcein 7 and 3 days prior to sacrifice. All specimens exposed distinctive green fluorescent calcein labels at both time points, whereas the red alizarin dye was more pronounced after 6 compared to 12 weeks healing time. In general, two directions of bone formation occurred: starting from defect margin and from the nasal septum. The control group showed a homogenous ossification, which led to cone-like shaped areas of cancellous bone, beginning on the former defect margin (Figures 5A,B). After 12 weeks maturation into lamellar bone was observable. In cases of scaffold insertion, a green auto-fluorescence of the biomaterial, as well as bone formation toward the scaffold were visible. After 6 weeks, a bone-to-scaffold contact was detected in some cases of scaffold A (Figures 5C,D). In this situation, the labeling was interrupted in the area of contact. Scaffold A + rMSC exposed a comparably smaller fluorescence labeling, indicating a reduced rate of bone formation at the time point of marker application. The insertion of scaffold B also led to an osteogenesis starting at the defect margins. Compared to scaffold A the bone-to-scaffold-contact was rarely visible in scaffold B. Between 6 and 12 weeks, both groups of scaffold B (with and w/o rMSC) increased bone formation, but to a smaller extent compared to scaffold A. In cases of suboptimal scaffold position, e.g., angular position, the bone formation was more pronounced on the defect site, which was closer to the biomaterial. If the scaffold was inserted too far into a cranial position, the bone grew underneath.

#### Histomorphology: Masson-Goldner Trichrome Staining

**FIGURE 6:**
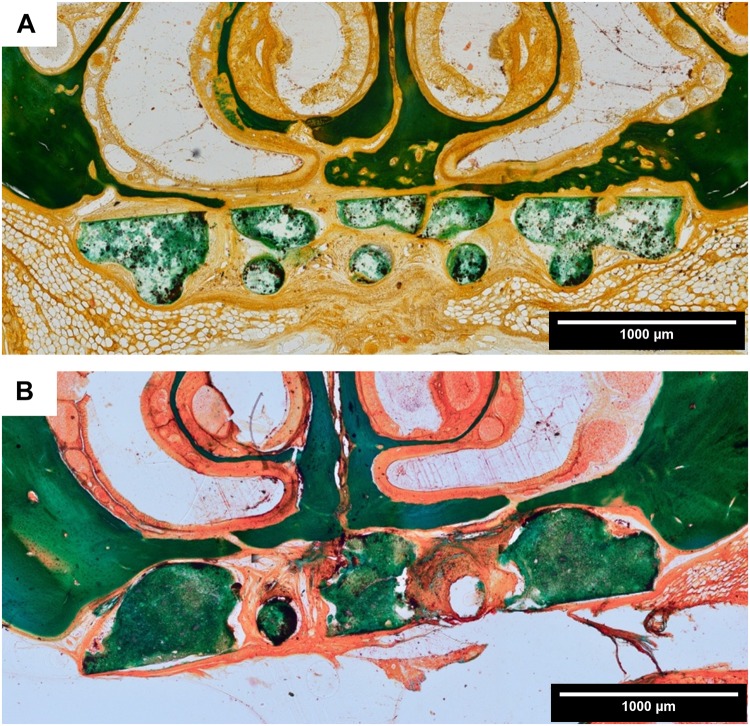
Histomorphology: representative images of Masson-Goldner trichrome staining in coronal sections. **(A)** Scaffold A after 6 weeks healing time; **(B)** scaffold A after 12 weeks healing time (both 10 × 10 magnification). There is an ongoing bone formation detectable, whereby the scaffold acts as a guiding structure for the cancellous bone. The scaffold did not show signs of resorption at the end of the healing period.

All animals showed a healed defect site and no oronasal fistulae were detected. The discrimination between host bone, which was dense lamellar bone, and newly formed bone was easily detectable due to morphological characteristics. Irrespective of the experimental group, after 6 weeks cone-like shaped cancellous bone grew into direction of the defect center and also osteoid structures were detectable at the tip of the cone. With ongoing healing time, bone maturation occurred, resulting in a more or less pronounced osseous bridging of the defect. In the control group the defect was filled with soft tissue and no osseous bridging took place. Groups with the scaffold types A and B primarily showed a fibrous integration of the defect, however, on the scaffold surface itself cancellous bone grew toward the defect center and the biomaterial acted as a guiding structure (Figure 6A). The shape of the newly formed bone was determined by the scaffold geometry. During the healing period, cancellous bone matured into lamellar bone (Figure 6B). In comparison to cell-free scaffolds of type A, rMSC-colonized scaffolds showed only negligible differences in bone formation. A bone-to-scaffold-contact occurred more frequently for the non-enriched scaffold compared to the scaffold + rMSC. Defects augmented with scaffold B exposed a smaller amount of newly formed bone compared to scaffold A, irrespective of rMSC colonization. In all groups, no resorption of the biomaterial was visible and no complete osseous healing of the defect was detected. Bone formation was especially impeded, in case scaffolds and their pores were covered by a thick layer of fibrous tissue.

#### Histomorphometry

##### Remaining defect width [mm]

**FIGURE 7:**
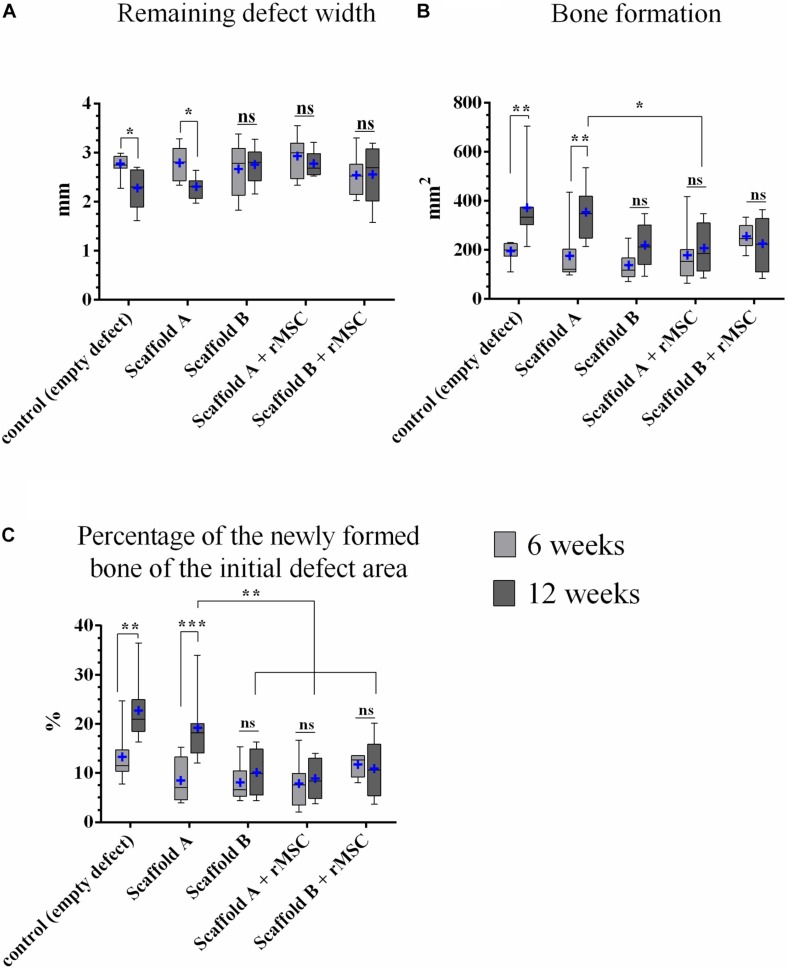
Histomorphometric analysis. **(A)** Remaining defect width, **(B)** bone formation, and **(C)** percentage of the newly formed bone related to the initial defect area (*n* = 8; median and minimum/maximum values; mean is marked by +; *p < 0.05, **p < 0.01, ***p < 0.001). All mean values and standard deviations are shown in Supplementary Table S1.

The remaining defect width decreased in all experimental groups in comparison to the initial defect’s width and with ongoing healing time. The reduction of the defect width from 6 to 12 weeks was statistically significant for control and scaffold A groups (Figure 7A). This parameter showed no statistically significant differences, comparing all experimental groups.

##### Bone formation in the defect area [mm^2^]

Osseous defect healing was detectable in all groups, but the extent differed (Figure 7B). After 6 weeks, the highest values were measured for scaffold B + rMSC, followed by the control group, scaffold A + rMSC, scaffold A and scaffold B; however, these differences were not significant. After 12 weeks, the results changed as the control and scaffold A exposed the largest areas of bone formation – only for this two groups the increase from 6 to 12 weeks was statistically significant (control: *p* = 0.002; scaffold A *p* = 0.001). Comparing all groups among each other after 12 weeks, the following result was found: the control group showed significantly more bone formation compared to scaffold A + rMSC (*p* = 0.028), scaffold B (*p* = 0.021) and scaffold B + rMSC (*p* = 0.031); bone formation in the scaffold A group was in the same range as those in the control group. Also scaffold A led to more osseous healing within the defect area compared to scaffold A + rMSC after 12 weeks (*p* = 0.048); in case of scaffold B, no significant differences between the groups with and without rMSC were observed. These findings were supported by measurements of the closest distance between scaffold and ingrowing bone, which was decreasing with ongoing healing time (Supplementary Figure S1 and Supplementary Table S2).

##### Percentage of the newly formed bone related to the initial defect area [%]

If the area of bone formation was calculated against the particular initial defect area, the values were in accordance to the results of the bone area (Figure 7C). After 6 weeks, the control group exposed an osseous defect healing of 13.2% (mean) with a significant increase to 22.5% at the end of the study. Also scaffold A showed a significantly increasing bone formation from 8.2 to 19.0%. The other groups had a smaller percentage of bone formation and no significant differences occurred between 6 and 12 weeks within the groups (Figure 7C). After 12 weeks, the control group showed significantly higher values of bone formation compared to scaffold A + rMSC, scaffold B, and scaffold B + rMSC (all *p* < 0.001). Also 19.0% of scaffold A was significantly more than 10.2% of scaffold B (*p* = 0.02), 10.8 of scaffold A + rMSC (*p* = 0.035) or 8.75% of scaffold A + rMSC (*p* = 0.002).

### Fabrication of Patient-Specific, CPC-Based Bone Grafts for Alveolar Cleft Osteoplasty: Proof of Concept

**FIGURE 8:**
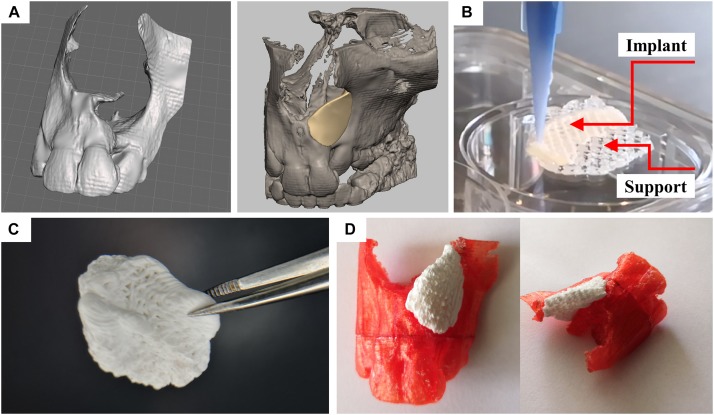
Design and fabrication process of a patient-specific implant consisting of a calcium phosphate cement. **(A)** Three-dimensional reconstruction of a CT scan of a patient and designed model of a perfectly fitting implant. **(B)** Fabrication process by multichannel 3D plotting of a sacrificial ink and the CPC. **(C)** Photograph of the implant, after the sacrificial ink was washed away. **(D)** The hardened implant fits perfectly into the defect of the patient. Red: Plastic model of the maxilla with incisors, white: 3D plotted CPC implant.

For the application in patients, the fabrication process has to be scaled up and the shape of the implant has to be tailored to the patient-individual cleft geometry. In a recent study, we showed that multichannel 3D plotting of CPC and a methylcellulose sacrificial ink enables the fabrication of complex shaped constructs exhibiting anatomical relevant features including convex and concave curvature at surfaces (Ahlfeld et al., 2018b). Herein, we successfully transferred that principle to the full additive manufacturing process chain from clinical three-dimensional imaging to the fabrication of a perfectly fitting patient-specific implant (Figure 8). In the first step, anonymized CT data of patients with an alveolar cleft were reconstructed using the software Dornheim segmenter achieving a 3D rendered model of the damaged maxilla of the patient (Figure 8A). The 3D model was then further processed (i.e., closing holes or smoothing of the polygonal mesh) with the software tool Geomagic Studio. Finally, the exact defect area was identified in order to enable the design of a patient-specific implant. In this case, the modeling of the patient specific shaped implant was performed utilizing Geomagic Freeform. This software offers the possibility of haptic interaction. This enabled us to fit the implant model into the given geometry of the upper jaw in the best possible way (Figure 8A). The implant was designed according to the internal and external geometry of the contralateral orofacial anatomy represented by the CT data. The dimension of the outer barrier was chosen to entirely cover the defect’s side edge-to-edge with the surrounding jaw bone.

The support structure, being required for the production of real three-dimensional shapes with overhanging structures, was designed as a negative form of the partially convex shape of the implant. The resulting *3mf* file, capable to distinguish the two materials (methylcellulose as sacrificial ink and CPC as biomaterial ink for the implant) was transferred to the multichannel plotter software being used for the production of the *in vivo* scaffolds. Both inks could be fabricated in layer-wise structure achieving the desired geometry. The angle between deposited layers was chosen at 60° according to the obtained results for ideal tissue integration. After hardening of the CPC, the methylcellulose could easily be dissolved without affecting the implants’ integrity and geometry (Figures 8B,C). During the mild post-processing of the CPC (setting in water-saturated atmosphere), no swelling or shrinking of the plotted scaffolds occurred. This allowed perfect fitting of the CPC implant into the defect site, as demonstrated in a partial thermoplastic model of the maxilla, fabricated based on the reconstructed CT data by fused filament fabrication (Figure 8D).

## Discussion

### 3D Plotting – Impact of Geometry

The study hypothesis was, that “The application of a 3D plotted bone graft into an artificial maxillary bone defect leads to a significant reduction of the defect width after 12 weeks.” This could be confirmed for the scaffold A group. Crucially, in this experimental group, a significant ongoing bone formation was measurable, which led to a reduction of the defect width (significant between 6 and 12 weeks healing time), confirming that 3D plotting of bone grafts is a promising approach for application in maxillofacial surgery and especially for the treatment of alveolar clefts. To evaluate the defect model itself, an empty defect was compared and significant bone formation was also shown. However, it must be stressed, that only the scaffold groups have clinical relevance for the application in patients, because clinically there is a strong recommendation for defect augmentation. Alveolar cleft osteoplasty is performed to build up a sufficient bone volume into the former cleft area, which is filled by fibrous tissue and doesn’t enable an eruption of e.g., the permanent canine. Besides the positive effect on the tooth eruption the surgery closes oronasal fistulas, which can lead to oronasal fluid reflux during drinking or eating. If the alveolar osteoplasty is not performed a disadvantage in maxillary growing occurs and the deficiency in transversal and anterior-posterior jaw development causes facial asymmetries and interferences in dental occlusion. After 12 weeks, scaffold A led to a significant higher percentage of bone tissue within the defect area compared to scaffold B. The hypothesis has to be rebutted for the experimental group of scaffold B, as the bone formation and reduction of the defect width did not reach statistical significance after 12 weeks. Thus, it can be concluded, that the pore geometry of an applied bone graft has a considerable impact on the treatment of artificial alveolar clefts.

Extrusion-based additive manufacturing techniques (3D plotting and fused deposition modeling) allow high control over the internal pore geometry of scaffolds which was demonstrated to influence both, mechanical and biological properties (Hutmacher et al., 2001; Obregon et al., 2015; Kelly et al., 2018). For example, pore gradients in an osteochondral tissue model consisting of printed poly-ε-caprolactone scaffolds showed enhanced osteogenic differentiation of hMSC and expression of bone markers for a 15° layer orientation compared to 90° layer orientation, but enhanced chondrogenic differentiation of hMSC in the reverse case (Luca et al., 2016). In addition, Ostrowska et al. (2016) investigated the differentiation of hMSC on printed PCL scaffolds *in vitro* and observed differences in the ALP expression depending on the layer orientation. Confirming our findings, also in this study the layer orientations of 30° and 60° did not influence the ALP activity significantly (Ostrowska et al., 2016). Almeida et al. (2014) compared 3D printed chitosan scaffolds with 90° and 45° layer orientation, and thus different pore geometries, and evidenced an influence of these pores on the immune response by measuring different levels of TNF-α and Interleukin 12/23 expression. Likewise, ceramic scaffolds can be produced by extrusion printing and pore geometry was demonstrated to play a crucial role on both, mechanics and bone formation *in vivo* (Roohani-Esfahani et al., 2016; Entezari et al., 2019). For plotted CPC constructs, scaffolds with 90° (Moussa et al., 2015; Barba et al., 2017; Baranowski et al., 2018; Reitmaier et al., 2018) and 60° (Ahlfeld et al., 2019) lay-down patterns were investigated *in vivo* and bone formation could be evidenced in every study. However, to the best knowledge of the authors, a comparative study with CPC scaffolds was not performed so far and the influence of pore geometry is still not answered satisfactorily.

In our approach, the layer orientation of 60° was superior compared to the layer orientation of 30°. Crucially, the layer orientation did not just influence the inner pore structure, but also the pores at the outer periphery of the CPC scaffolds (caused by the meanders between the strands, see Figure 2) which are in direct contact with the host bone. These outer pores are crucial for ingrowing bone, which utilizes the scaffold’s surface as guiding structure, and therefore, their size and shape might influence this process.

Another aspect of geometry has to be considered for comprehension of the *in vivo* results: the contact between scaffold and osseous defect margin was found to depend on the scaffold orientation. During surgery, it was easy to place the scaffolds A or B in the bone defect and clinically the fitting was acceptable in both groups; no unwanted movement of the scaffold or gaps occurred. Retrospectively we observed a relevance of the placement of the bone grafts with the “right site” into the defect: the scaffolds exhibited a smooth, flattened site due to a layer deformation which occurred in the first layer due to the contact with the plotting stage. In layer 2–4, the CPC strands stayed in round shape and the upper site of the scaffold displayed convex and concave surfaces. As it did not clinically matter which site was turned into nasal or oral direction, the position of the scaffold was randomly chosen. However, analysis of the micro-CT and histological images revealed that the fitting was better, showing a minimal distance between bone and scaffold, if the smooth (bottom) site was placed into oral direction. In this case, the scaffold represented a guiding structure for newly formed bone and the closure of a critical size defect became probable. Thus, both, inner and outer geometry of the 3D plotted bone scaffolds played a crucial role on the osseous healing.

Compared to our previous studies conducted in the same defect model (Korn et al., 2014, 2017), the 3D plotted CPC scaffolds were superior to granular materials with respect to fitting into the defect. In both studies, the initial clinical fitting of the bone grafts, hydroxyapatite-beta-tricalcium phosphate with a granular structure (BONIT matrix^®^) (Korn et al., 2014) and hydroxyapatite granules embedded in a fast resorbing collagen matrix (BioOss^®^ Collagen) (Korn et al., 2017), were good and there was clinically no distance between defect margin and scaffold. However, in case of BioOss^®^ Collagen, the initial compact structure of the biomaterial was dispersed into smaller granula and in both studies, fibrous encapsulation of the particles was observed, probably because of their instable position within the defect; accordingly, no osseous integration or complete defect ossification occurred. If the granulae were located adjacent to the host bone osseous integration took place leading to the conclusion, that a more stable, defined structure of the scaffold is required for further studies. Therefore, plotted CPC scaffolds were chosen and it can be concluded from the current results that after 12 weeks the scaffold position was more predictable compared to the previously tested materials. Although the problem of dislocation did not occur anymore, there is still improvement required: a partial degradation of the scaffold is necessary as the newly formed bone should replace the bone graft after a defined period of time leading to a complete defect ossification.

One essential aspect of future research on geometrical issues could be the realization of anisotropic artificial scaffold structures as they appear in native bone. Today, this can be achieved via variation of the strand-to-strand distance and porosity. Our future aim will be the generation of density gradients in spatial definition within the fabrication process without the need of changing the total number of strands by implementing alternative printing path patterns, which replace the usual printing paths of straight lines between two points.

### Tissue Engineering – Impact of Cell Pre-colonization

Considering the great potential of the tissue engineering approach (as alternative to autologous bone graft), we investigated whether a pre-colonization of the plotted CPC scaffolds with rMSC enhances bone formation. Compared to our previous studies, which were conducted with the same defect model using commercially available and clinically established materials, the findings presented in the present study showed distinctly different insights into scaffold-bone interactions (Korn et al., 2014, 2017). In the first study, resorbable bone grafts consisting of a synthetic nanocrystalline hydroxyapatite-beta-tricalcium phosphate mixture (BONIT matrix^®^), which had a granular structure, were colonized with undifferentiated as well as osteogenically differentiated rMSC prior to implantation. The smallest defect, and therefore strongest new bone formation, was observed in the group using bone grafts with undifferentiated rMSC (remaining defect width after 6 weeks: 2.39 mm ± 0.23 mm). Compared to the non-enriched scaffolds, which exposed a remaining defect width of 2.70 mm after 6 weeks, the pre-colonized scaffold was superior, but this was not statistically significant (Korn et al., 2014). Also in combination with a bovine hydroxyapatite granule-collagen graft (BioOss^®^ Collagen), the undifferentiated rMSC were more effective compared to the colonization with osteogenic differentiated rMSC. Nevertheless, the non-enriched scaffold without rMSC pre-colonization finally exposed the significantly smallest remaining defect width after 12 weeks (Korn et al., 2017). Based on the previous results, only undifferentiated rMSC were chosen for this experimental study. It was surprising, that in the current study both, cells on scaffold A or B, did not show this positive effect. Considering these diverse and partly contradictory results of preclinical studies, the impact of rMSC onto the healing in the alveolar cleft remains unclear. Also in a clinical trial with 20 patients, Hermund et al. (2012) observed no significant difference of bone formation in the maxilla between bone scaffolds with or without cells. Ricci et al. (2012) demonstrated that in cases of vital bone adjacent to the defect, it may not require a cell-seeded scaffold for complete defect ossification. They accentuated the inherent problems with cell-seeding strategies and recommended in case of bone repair osteoconductive materials like CPC or ceramics in shape of highly organized 3D scaffolds, that guide the newly formed bone across the bone defect also without additional cell colonization (Ricci et al., 2012). In contrast, cell-seeded bone grafts were shown to induce better bone formation than cell-free equivalents in other studies (Eniwumide et al., 2007; Korn et al., 2014). Although our *in vitro* characterization clearly demonstrated that rMSC were able to proliferate and differentiate, no significantly increased bone formation was observed *in vivo*. In the light of these observations and the fact that especially after seeding of scaffolds with clinically relevant dimensions the cells often suffer from an insufficient supply after implantation due to the lack of vascularization (Jaklenec et al., 2012), the classical tissue engineering approach is questionable. A promising alternative for further studies might be the concept of *in situ* tissue engineering which envisages the recruitment of host stem and progenitor cells to the defect site by chemoattractive factors released from the scaffold (Ko et al., 2013).

### 3D Plotted CPC Scaffolds as Potential Material for Alveolar Cleft Osteoplasty

Classical fabrication methods of bone grafts are limited in their clinical application with respect to patient-individual treatment, however, additive manufacturing seems to be a promising technique to produce implants for alveolar defects. In contrast to other bone defects, it is mandatory to use biodegradable materials for the treatment of alveolar clefts, as the patients are children in growth and non-degradable materials would possibly influence local growth. This might be the reason, why, up to now, only a few materials were tested for the treatment of such defects and most studies concentrate on secondary bone grafting (Seifeldin, 2016). These materials include hydroxyapatite granules (Korn et al., 2014, 2017), bio-ceramics coated with bovine collagen and dipyridamole (Lopez et al., 2018), tricalcium phosphates (TCP) (Janssen et al., 2014; Berger et al., 2015), demineralized bone matrix (Francis et al., 2013), thermoplastic poly-ε-caprolactone (PCL) (Ahn et al., 2018; Puwanun et al., 2018) or a β-TCP/PCL composite (Raúl et al., 2019). In a recent review, Martín-del-Campo et al. (2019) suggested several biomaterial-based strategies which have high potential for cleft repair. Herein, we introduced a self-setting CPC for the three-dimensional fabrication of cleft alveolar osteoplasties offering several advantages. CPC can be plotted into complex shaped scaffolds revealing high shape fidelity and accuracy and plotted structures do not change dimensions while post-processing (Lode et al., 2014; Ahlfeld et al., 2018b). Plotted CPC scaffolds are highly biocompatible (Lode et al., 2014; Raymond et al., 2018; Ahlfeld et al., 2019) and can be biofunctionalized to enhance their biological performance, for example by growth factor loading (Akkineni et al., 2015; Ahlfeld et al., 2017, 2019; Baranowski et al., 2018) or even by integration of CPC into bioprinting with spatially defined cells (Ahlfeld et al., 2018a). For this first approach, we utilized pure CPC, which is clinically approved and answers general questions of this approach. CPC plotting can be miniaturized achieving filigree structures (Ahlfeld et al., 2017) as used in the scaffolds of this study. Furthermore, we could show in this work, that CPC scaffolds can be fabricated in clinically relevant geometries to fill real alveolar defects. As the CPC formulation transforms into nanocrystalline, bioresorbable hydroxyapatite (Heinemann et al., 2013), the scaffolds can be resorbed by osteoclasts (Bernhardt et al., 2014) and are integrated into the natural bone remodeling process [lasting about 200 days until 2 years in the human body (Eriksen, 2010)], which we consider as advantageous for the healing process. However, our investigations did not confirm visible resorptions of the scaffolds, which is related to the general slow degradation of HA forming cements (Thormann et al., 2013). This might be overcome by composites consisting of CPC and fast degrading biomaterials such as mesoporous bioactive glass, which were shown to enhance the degradation of such constructs distinctly and even to promote osteogenesis (Schumacher et al., 2017; Richter et al., 2019) or by a novel oil-based calcium doped magnesium phosphate cement which demonstrated enhanced degradation (Ewald et al., 2019).

First clinical studies evaluated the effects of printed bone grafts on osseous healing in alveolar cleft patients. Ahn et al. (2018) fabricated cleft osteoplasties consisting of PCL by fused filament fabrication. In an initial case study with a 10-years-old patient, the scaffold promoted bone formation; after 6 months 45% of the defect was filled by ingrowing bone (Ahn et al., 2018). A similar approach utilizing a PCL-β-tricalcium phosphate composite evidenced the potential of bioresorbable bone grafts for cleft palates (Raúl et al., 2019). In the light of these results, the approach investigated in this study utilizing 3D plotted CPC scaffolds demonstrates very high potential for further research and clinical application (approximately 20% bone formation after 3 months). Nevertheless, more studies are needed to ascertain the long-term clinical results of alveolar cleft reconstruction using tissue engineered and additively manufactured bone grafts (Wu et al., 2018).

### Limitations of the Study

The complexity of a congenital human alveolar cleft or palate cannot be fully displayed by any pre-clinical model. Therefore, experimental studies are limited to artificial bone defects in different models. An established small animal model is the rat. This is due to the fact that a cleft-like defect can be prepared surgically in its maxillary bone (Mehrara et al., 2000; Mostafa et al., 2014). If cells should be transferred inter-individually, the selection of an inbreeding breed, like Lewis rats, avoid immunological problems. Advantage of the chosen animal model, is the possibility to insert bone grafts intraorally. However, physiological interactions with the oral microorganism as well as chewing forces can occur. One disadvantage of the oral defect in a small animal model is the inability of a complete wound rest. For this reason, rats were fed a soft diet to reduce micromovements. In a clinical setting, patients can be nourished by a nasal gastral tube to enable a proper initial wound rest. A limitation of this study may be the fact, that the results after 6 and 12 weeks are not gained for the same animal by e.g., intravital micro-CT imaging. The reason was, that the imaging properties of CPC did not enable an accurate quantification of the bone formation and therefore *ex vivo* histology was essential to evaluate the defect healing. Also the number of specimens per defect was limited due to the sawing- and grinding method, but average 3.2 samples are acceptable to quantify bone formation *in vivo* (Bernhardt et al., 2012). After identifying a promising bone graft in a small animal model the evaluation in a larger model would be necessary. Suitable models are dogs (Zhang et al., 2011), primate (Boyne, 2001), and pigs (Caballero et al., 2015). From a clinical point of view, the tissue engineered bone grafts should be compared with autologous bone graft and not only with an empty control defect. This however is only feasible in large animal models (Pourebrahim et al., 2013). Assuming application of autologous bone grafts in the rat model, e.g., from the femur, the fixation into the defect would be challenging. Reasons are the low height of the defect (less than 0.5 mm) and the open connection into the nasal cavity, which would lead to an initial dislocation of the bone granules.

## Conclusion

3D plotting of CPC is suitable for the fabrication of scaffolds, which are fitting exactly in an artificial alveolar defect. We could show that the fabrication of such scaffolds can also be translated toward clinical indications and real defect geometries of patients. The pore geometry influences bone formation significantly; a 60° strand rotation leading to triangular-shaped pores performed significantly better than a 30° strand rotation. In this study, an additional colonization with undifferentiated rMSC did not result in an increased bone formation. Furthermore, no signs of scaffold degradation occurred, pointing out the necessity of further material development like modification of the cement matrix with porogens or adapting the composition toward more soluble phases. The creation of a sufficient 3D printed and tissue-engineered bone graft for alveolar cleft osteoplasty could preserve patients from donor site morbidity. Further studies will focus on improving the degradation properties of the 3D plotted bone grafts by using CPC modifications as well as on the stable fixation in the defect area which might increase the local bone formation. With regard to clinical application, the behavior of a new bone graft should also be tested in alveolar defects of large animal models.

## Data Availability Statement

The datasets generated for this study are available on request to the corresponding author.

## Ethics Statement

The animal study was reviewed and approved by Commission for Animal Studies at the District Government Dresden, Germany (DD24-5131/354/26).

## Author Contributions

PK: study design, surgery, histology, evaluation micro-CT, and manuscript editing. TA: design and preparation of the 3D printed scaffolds, *in vitro* evaluation, and manuscript editing. FL: histomorphology and histomorphometry, evaluation of micro-CT images, statistics, and manuscript editing. DK: design and preparation of the patient specific implant, and manuscript editing. PS: design and preparation of the patient specific implant. RS: design of the patient specific implant. WP: surgery and manuscript editing. AF: surgery. MR: planning and realization micro-CT evaluation. UR: statistics. BS: study design and manuscript editing. AL: study design, *in vitro* evaluation, and manuscript editing. GL: study design. MG: study design and manuscript editing.

## Conflict of Interest

The authors declare that the research was conducted in the absence of any commercial or financial relationships that could be construed as a potential conflict of interest.

## Supplementary Material

FIGURE S1 Histomorphometric analysis. Closest distance between scaffold and defect margin. **(A)** Initial distance at time point of implant placement. **(B)** Final distance at the end of the study after 6 or 12 weeks (median and minimum/maximum values, mean is marked by +, **p* < 0.05, ***p* < 0.01).

TABLE S1 Measurements in the histomorphological images (mean ± standard deviation) for the remaining defect width, bone formation and percentage of the newly formed bone related to the initial defect area after 6 and 12 weeks.

TABLE S2 Measurements in the histomorphological images (mean ± standard deviation) for the initial and final distance between bony defect margin and scaffold margin after 6 and 12 weeks.

## References

[B1] Ahlfeld T. Akkineni A. R. Förster Y. Köhler T. Knaack S. Gelinsky M. (2017). Design and fabrication of complex scaffolds for bone defect healing: combined 3D plotting of a calcium phosphate cement and a growth factor-loaded hydrogel. *Ann. Biomed. Eng.* 45 224–236. 10.1007/s10439-016-1685-4 27384939

[B2] Ahlfeld T. Doberenz F. Kilian D. Vater C. Korn P. Lauer G. (2018a). Bioprinting of mineralized constructs utilizing multichannel plotting of a self-setting calcium phosphate cement and a cell-laden bioink. *Biofabrication* 10:045002. 10.1088/1758-5090/aad36d 30004388

[B3] Ahlfeld T. Köhler T. Czichy C. Lode A. Gelinsky M. (2018b). A methylcellulose hydrogel as support for 3D plotting of complex shaped calcium phosphate scaffolds. *Gels* 4:68. 10.3390/gels4030068 30674844 PMC6209251

[B4] Ahlfeld T. Schuster F. P. Förster Y. Quade M. Akkineni A. R. Rentsch C. (2019). 3D plotted biphasic bone scaffolds for growth factor delivery: biological characterization in vitro and in vivo. *Adv. Healthc. Mater.* 8:1801512. 10.1002/adhm.201801512 30838778

[B5] Ahn G. Lee J.-S. Yun W.-S. Shim J.-H. Lee U.-L. (2018). Cleft alveolus reconstruction using a three-dimensional printed bioresorbable scaffold with human bone marrow cells. *J. Craniofac. Surg.* 29 1880–1883. 10.1097/SCS.0000000000004747 30028404

[B6] Akkineni A. R. Luo Y. Schumacher M. Nies B. Lode A. Gelinsky M. (2015). 3D plotting of growth factor loaded calcium phosphate cement scaffolds. *Acta Biomater.* 27 264–274. 10.1016/j.actbio.2015.08.036 26318366

[B7] Almeida C. R. Serra T. Oliveira M. I. Planell J. A. Barbosa M. A. Navarro M. (2014). Impact of 3-D printed PLA- and chitosan-based scaffolds on human monocyte/macrophage responses: unraveling the effect of 3-D structures on inflammation. *Acta Biomater.* 10 613–622. 10.1016/j.actbio.2013.10.035 24211731

[B8] Baranowski A. Klein A. Ritz U. Götz H. Mattyasovszky S. G. Rommens P. M. (2018). Evaluation of bone sialoprotein coating of three-dimensional printed calcium phosphate scaffolds in a calvarial defect model in mice. *Materials* 11:2336. 10.3390/ma11112336 30469365 PMC6267578

[B9] Barba A. Diez-Escudero A. Maazouz Y. Rappe K. Espanol M. Montufar E. B. (2017). Osteoinduction by foamed and 3D-printed calcium phosphate scaffolds: effect of nanostructure and pore architecture. *ACS Appl. Mater. Interfaces* 9 41722–41736. 10.1021/acsami.7b14175 29116737

[B10] Berger M. Probst F. Schwartz C. Cornelsen M. Seitz H. Ehrenfeld M. (2015). A concept for scaffold-based tissue engineering in alveolar cleft osteoplasty. *J. Craniomaxillofac. Surg.* 43 830–836. 10.1016/j.jcms.2015.04.023 26027868

[B11] Bernhardt A. Schumacher M. Gelinsky M. (2014). Formation of osteoclasts on calcium phosphate bone cements and polystyrene depends on monocyte isolation conditions. *Tissue Eng. C. Met.* 21 160–170. 10.1089/ten.tec.2014.0187 24919531

[B12] Bernhardt R. Kuhlisch E. Schulz M. C. Eckelt U. Stadlinger B. (2012). Comparison of bone-implant contact and bone-implant volume between. *Eur. Cell Mater.* 23 237–247. discussion 247–248 22492016 10.22203/ecm.v023a18

[B13] Boehm K. S. Al-Taha M. Morzycki A. Samargandi O. A. Al-Youha S. LeBlanc M. R. (2018). Donor site morbidities of iliac crest bone graft in craniofacial surgery: a systematic review. *Ann. Plast. Surg.* 83 352–358. 10.1097/SAP.0000000000001682 30562201

[B14] Boyne P. J. (2001). Application of bone morphogenetic proteins in the treatment of clinical oral and maxillofacial osseous defects. *J. Bone Joint Surg. Am.* 83-A (Suppl. 1) (Pt 2) 146–150. 11314792

[B15] Caballero M. Morse J. C. Halevi A. E. Emodi O. Pharaon M. R. Wood J. S. (2015). Juvenile swine surgical alveolar cleft model to test novel autologous stem cell therapies. *Tissue Eng. Part C Methods* 21 898–908. 10.1089/ten.TEC.2014.0646 25837453 PMC4553376

[B16] Dixon M. J. Marazita M. L. Beaty T. H. Murray J. C. (2011). Cleft lip and palate: understanding genetic and environmental influences. *Nat. Rev. Genet.* 12 167–178. 10.1038/nrg2933 21331089 PMC3086810

[B17] Donath K. Breuner G. (1982). A method for the study of undecalcified bones and teeth with attached soft tissues. The Sage-Schliff (sawing and grinding) technique. *J. Oral Pathol.* 11 318–326. 10.1111/j.1600-0714.1982.tb00172.x 6809919

[B18] Eniwumide J. O. Yuan H. Cartmell S. H. Meijer G. J. de Bruijn J. D. (2007). Ectopic bone formation in bone marrow stem cell seeded calcium phosphate scaffolds as compared to autograft and (cell seeded) allograft. *Eur. Cell Mater.* 14 30–38. 10.22203/eCM.v014a03 17674330

[B19] Entezari A. Roohani I. Li G. Dunstan C. R. Rognon P. Li Q. (2019). Architectural design of 3D printed scaffolds controls the volume and functionality of newly formed bone. *Adv. Healthc. Mater.* 8:1801353. 10.1002/adhm.201801353 30536610

[B20] Eriksen E. F. (2010). Cellular mechanisms of bone remodeling. *Rev. Endocr. Metab. Disord.* 11 219–227. 10.1007/s11154-010-9153-1 21188536 PMC3028072

[B21] Ewald A. Kreczy D. Brückner T. Gbureck U. Bengel M. Hoess A. (2019). Development and bone regeneration capacity of premixed magnesium phosphate cement pastes. *Materials* 12:2119. 10.3390/ma12132119 31266228 PMC6651064

[B22] Fedorovich N. E. De Wijn J. R. Verbout A. J. Alblas J. Dhert W. J. A. (2008). Three-dimensional fiber deposition of cell-laden, viable, patterned constructs for bone tissue printing. *Tissue Eng. Part A* 14 127–133. 10.1089/ten.a.2007.0158 18333811

[B23] Feinberg S. E. Weisbrode S. E. Heintschel G. (1989). Radiographic and histological analysis of tooth eruption through calcium phosphate ceramics in the cat. *Arch. Oral Biol.* 34 975–984. 10.1016/0003-9969(89)90055-1 2558643

[B24] Francis C. Mobin S. S. Lypka M. Rommer E. Yen S. Urata M. (2013). rhBMP-2 with a demineralized bone matrix scaffold versus autologous iliac crest bone graft for alveolar cleft reconstruction. *Plast. Reconstr. Surg.* 131 1107–1115. 10.1097/PRS.0b013e3182865dfb 23385986

[B25] Guo J. Li C. Zhang Q. Wu G. Deacon S. A. Chen J. (2011). Secondary bone grafting for alveolar cleft in children with cleft lip or cleft lip and palate. *Cochrane Database Syst. Rev.* 6:CD008050. 10.1002/14651858.CD008050.pub2 21678372

[B26] Heinemann S. Rössler S. Lemm M. Ruhnow M. Nies B. (2013). Properties of injectable ready-to-use calcium phosphate cement based on water-immiscible liquid. *Acta Biomater.* 9 6199–6207. 10.1016/j.actbio.2012.12.017 23261920

[B27] Hermund N. U. Stavropoulos A. Donatsky O. Nielsen H. Clausen C. Reibel J. (2012). Reimplantation of cultivated human bone cells from the posterior maxilla for sinus floor augmentation. Histological results from a randomized controlled clinical trial. *Clin. Oral Implants Res.* 23 1031–1037. 10.1111/j.1600-0501.2011.02251.x 22092973

[B28] Hutmacher D. W. Schantz J. T. Lam C. X. F. Tan K. C. Lim T. C. (2007). State of the art and future directions of scaffold-based bone engineering from a biomaterials perspective. *J. Tissue Eng. Regen. Med.* 1 245–260. 10.1002/term.24 18038415

[B29] Hutmacher D. W. Schantz T. Zein I. Ng K. W. Teoh S. H. Tan K. C. (2001). Mechanical properties and cell cultural response of polycaprolactone scaffolds designed and fabricated via fused deposition modeling. *J. Biomed. Mater. Res.* 55 203–216. 10.1002/1097-4636(200105)55:2<203::AID-JBM1007>3.0.CO;2-7 11255172

[B30] Jaklenec A. Stamp A. Deweerd E. Sherwin A. Langer R. (2012). Progress in the tissue engineering and stem cell industry “are we there yet?”. *Tissue Eng. Part B Rev.* 18 155–166. 10.1089/ten.teb.2011.0553 22220809

[B31] Janssen N. G. Weijs W. L. J. Koole R. Rosenberg A. J. W. P. Meijer G. J. (2014). Tissue engineering strategies for alveolar cleft reconstruction: a systematic review of the literature. *Clin. Oral Investig.* 18 219–226. 10.1007/s00784-013-0947-x 23430342

[B32] Kelly C. N. Miller A. T. Hollister S. J. Guldberg R. E. Gall K. (2018). Design and structure-function characterization of 3D printed synthetic porous biomaterials for tissue engineering. *Adv. Healthc. Mater.* 7:1701095. 10.1002/adhm.201701095 29280325

[B33] Kim H. D. Amirthalingam S. Kim S. L. Lee S. S. Rangasamy J. Hwang N. S. (2017). Biomimetic materials and fabrication approaches for bone tissue engineering. *Adv. Healthc. Mater.* 6:1700612. 10.1002/adhm.201700612 29171714

[B34] Ko I. K. Lee S. J. Atala A. Yoo J. J. (2013). In situ tissue regeneration through host stem cell recruitment. *Exp. Mol. Med.* 45:e57. 10.1038/emm.2013.118 24232256 PMC3849571

[B35] Korn P. Hauptstock M. Range U. Kunert-Keil C. Pradel W. Lauer G. (2017). Application of tissue-engineered bone grafts for alveolar cleft osteoplasty in a rodent model. *Clin. Oral Investig.* 21 2521–2534. 10.1007/s00784-017-2050-1 28101680

[B36] Korn P. Schulz M. C. Range U. Lauer G. Pradel W. (2014). Efficacy of tissue engineered bone grafts containing mesenchymal stromal cells for cleft alveolar osteoplasty in a rat model. *J. Craniomaxillofac. Surg.* 42 1277–1285. 10.1016/j.jcms.2014.03.010 24831850

[B37] Landers R. Pfister A. Hübner U. John H. Schmelzeisen R. Mülhaupt R. (2002). Fabrication of soft tissue engineering scaffolds by means of rapid prototyping techniques. *J. Mater. Sci.* 37 3107–3116. 10.1023/A:1016189724389 26991636

[B38] Liang F. Leland H. Jedrzejewski B. Auslander A. Maniskas S. Swanson J. (2018). Alternatives to autologous bone graft in alveolar cleft reconstruction: the state of alveolar tissue engineering. *J. Craniofac. Surg.* 29 584–593. 10.1097/SCS.0000000000004300 29461365

[B39] Lode A. Heiss C. Knapp G. Thomas J. Nies B. Gelinsky M. (2018). Strontium-modified premixed calcium phosphate cements for the therapy of osteoporotic bone defects. *Acta Biomater.* 65 475–485. 10.1016/j.actbio.2017.10.036 29107056

[B40] Lode A. Meissner K. Luo Y. Sonntag F. Glorius S. Nies B. (2014). Fabrication of porous scaffolds by three-dimensional plotting of a pasty calcium phosphate bone cement under mild conditions. *J. Tissue Eng. Regen. Med.* 8 682–693. 10.1002/term.1563 22933381

[B41] Lopez C. D. Witek L. Torroni A. Flores R. L. Demissie D. B. Young S. (2018). The role of 3D printing in treating craniomaxillofacial congenital anomalies. *Birth Defects Res.* 110 1055–1064. 10.1002/bdr2.1345 29781248 PMC6117201

[B42] Luca A. D. Lorenzo-Moldero I. Mota C. Lepedda A. Auhl D. Blitterswijk C. V. (2016). Tuning cell differentiation into a 3D scaffold presenting a pore shape gradient for osteochondral regeneration. *Adv. Healthc. Mater.* 5 1753–1763. 10.1002/adhm.201600083 27109461

[B43] Malda J. Visser J. Melchels F. P. Jüngst T. Hennink W. E. Dhert W. J. A. (2013). 25th anniversary article: engineering hydrogels for biofabrication. *Adv. Mater.* 25 5011–5028. 10.1002/adma.201302042 24038336

[B44] Martín-del-Campo M. Rosales-Ibañez R. Rojo L. (2019). Biomaterials for cleft lip and palate regeneration. *Int. J. Mol. Sci.* 20:2176. 10.3390/ijms20092176 31052503 PMC6540257

[B45] Mehrara B. J. Saadeh P. B. Steinbrech D. S. Dudziak M. Grayson B. H. Cutting C. B. (2000). A rat model of gingivoperiosteoplasty. *J. Craniofac. Surg.* 11 54–58. 10.1097/00001665-200011010-00010 11314101

[B46] Moroni L. Boland T. Burdick J. A. Maria C. D. Derby B. Forgacs G. (2018). Biofabrication: a guide to technology and terminology. *Trends Biotechnol.* 36 384–402. 10.1016/j.tibtech.2017.10.015 29137814

[B47] Mostafa N. Z. Doschak M. R. Major P. W. Talwar R. (2014). Reliable critical sized defect rodent model for cleft palate research. *J. Craniomaxillofac. Surg.* 42 1840–1846. 10.1016/j.jcms.2014.07.001 25150164

[B48] Moussa M. Carrel J.-P. Scherrer S. Cattani-Lorente M. Wiskott A. Durual S. (2015). Medium-term function of a 3D printed TCP/HA structure as a new osteoconductive scaffold for vertical bone augmentation: a simulation by BMP-2 activation. *Materials* 8 2174–2190. 10.3390/ma8052174 25350936

[B49] Nguyen P. D. Lin C. D. Allori A. C. Schachar J. S. Ricci J. L. Saadeh P. B. (2009). Scaffold-based rhBMP-2 therapy in a rat alveolar defect model: implications for human gingivoperiosteoplasty. *Plast. Reconstr. Surg.* 124 1829–1839. 10.1097/PRS.0b013e3181bf8024 19952639

[B50] Obregon F. Vaquette C. Ivanovski S. Hutmacher D. W. Bertassoni L. E. (2015). Three-dimensional bioprinting for regenerative dentistry and craniofacial tissue engineering. *J. Dent. Res.* 94 143S–152S. 10.1177/0022034515588885 26124216

[B51] Ostrowska B. Di Luca A. Moroni L. Swieszkowski W. (2016). Influence of internal pore architecture on biological and mechanical properties of three-dimensional fiber deposited scaffolds for bone regeneration. *J. Biomed. Mater. Res.* 104 991–1001. 10.1002/jbm.a.35637 26749200

[B52] Pfister A. Landers R. Laib A. Hübner U. Schmelzeisen R. Mülhaupt R. (2004). Biofunctional rapid prototyping for tissue-engineering applications: 3D bioplotting versus 3D printing. *J. Polym. Sci. A Polym. Chem.* 42 624–638. 10.1002/pola.10807

[B53] Poldervaart M. T. Wang H. van der Stok J. Weinans H. Leeuwenburgh S. C. G. Öner F. C. (2013). Sustained release of BMP-2 in bioprinted alginate for osteogenicity in mice and rats. *PLoS One* 8:e72610. 10.1371/journal.pone.0072610 23977328 PMC3747086

[B54] Pourebrahim N. Hashemibeni B. Shahnaseri S. Torabinia N. Mousavi B. Adibi S. (2013). A comparison of tissue-engineered bone from adipose-derived stem cell with autogenous bone repair in maxillary alveolar cleft model in dogs. *Int. J. Oral Maxillofac. Surg.* 42 562–568. 10.1016/j.ijom.2012.10.012 23219713

[B55] Pradel W. Lauer G. (2012). Tissue-engineered bone grafts for osteoplasty in patients with cleft alveolus. *Ann. Anat.* 194 545–548. 10.1016/j.aanat.2012.06.002 22776088

[B56] Puwanun S. Delaine-Smith R. M. Colley H. E. Yates J. M. MacNeil S. Reilly G. C. (2018). A simple rocker−induced mechanical stimulus upregulates mineralization by human osteoprogenitor cells in fibrous scaffolds. *J. Tissue Eng. Regen. Med.* 12 370–381. 10.1002/term.2462 28486747 PMC5836908

[B57] Raúl R. I. Nieves C. M. María R. L. L. Navarrete Amairany R. Olga F. S. M. L. (2019). Potential benefits from 3D printing and dental pulp stem cells in cleft palate treatments: an in vivo model study. *BJSTR* 16 11950–11953. 10.26717/BJSTR.2019.16.002831

[B58] Raymond S. Maazouz Y. Montufar E. B. Perez R. A. González B. Konka J. (2018). Accelerated hardening of nanotextured 3D-plotted self-setting calcium phosphate inks. *Acta Biomater.* 75 451–462. 10.1016/j.actbio.2018.05.042 29842972

[B59] Reitmaier S. Kovtun A. Schuelke J. Kanter B. Lemm M. Hoess A. (2018). Strontium(II) and mechanical loading additively augment bone formation in calcium phosphate scaffolds. *J. Orthop. Res.* 36 106–117. 10.1002/jor.23623 28574614

[B60] Ricci J. L. Clark E. A. Murriky A. Smay J. E. (2012). Three-dimensional printing of bone repair and replacement materials: impact on craniofacial surgery. *J. Craniofac. Surg.* 23 304–308. 10.1097/SCS.0b013e318241dc6e 22337431

[B61] Richter R. F. Ahlfeld T. Gelinsky M. Lode A. (2019). Development and characterization of composites consisting of calcium phosphate cements and mesoporous bioactive glass for extrusion-based fabrication. *Materials* 12:2022. 10.3390/ma12122022 31238538 PMC6630970

[B62] Roohani-Esfahani S.-I. Newman P. Zreiqat H. (2016). Design and fabrication of 3D printed scaffolds with a mechanical strength comparable to cortical bone to repair large bone defects. *Sci. Rep.* 6:19468. 10.1038/srep19468 26782020 PMC4726111

[B63] Schumacher M. Reither L. Thomas J. Kampschulte M. Gbureck U. Lode A. (2017). Calcium phosphate bone cement/mesoporous bioactive glass composites for controlled growth factor delivery. *Biomater. Sci.* 5 578–588. 10.1039/c6bm00903d 28154869

[B64] Seifeldin S. A. (2016). Is alveolar cleft reconstruction still controversial? (Review of literature). *Saudi Dent. J.* 28 3–11. 10.1016/j.sdentj.2015.01.006 26792963 PMC4688438

[B65] Silva Gomes Ferreira P. H. De Oliveira D. Duailibe De Deus C. B. Okamoto R. (2018). Evaluation of the different biomaterials used in alveolar cleft defects in children. *Ann. Maxillofac. Surg.* 8 315–319. 10.4103/ams.ams_140_17 30693253 PMC6327813

[B66] Thormann U. Ray S. Sommer U. ElKhassawna T. Rehling T. Hundgeburth M. (2013). Bone formation induced by strontium modified calcium phosphate cement in critical-size metaphyseal fracture defects in ovariectomized rats. *Biomaterials* 34 8589–8598. 10.1016/j.biomaterials.2013.07.036 23906515

[B67] Waite P. D. Waite D. E. (1996). Bone grafting for the alveolar cleft defect. *Semin. Orthod.* 2 192–196. 10.1016/s1073-8746(96)80014-4 9161288

[B68] Wu C. Pan W. Feng C. Su Z. Duan Z. Zheng Q. (2018). Grafting materials for alveolar cleft reconstruction: a systematic review and best-evidence synthesis. *Int. J. Oral Maxillofac. Surg.* 47 345–356. 10.1016/j.ijom.2017.08.003 28863859

[B69] Zhang D. Chu F. Yang Y. Xia L. Zeng D. Uludag H. (2011). Orthodontic tooth movement in alveolar cleft repaired with a tissue engineering bone: an experimental study in dogs. *Tissue Eng. Part A* 17 1313–1325. 10.1089/ten.TEA.2010.0490 21226625

