# Supplementary figures and images for: 3D Printing of Bone Grafts for Cleft Alveolar Osteoplasty – In vivo Evaluation in a Preclinical Model
Source: Front Bioeng Biotechnol. 2020 Mar 25;8:217. doi: 10.3389/fbioe.2020.00217 (PMC7109264; doi:10.3389/fbioe.2020.00217)

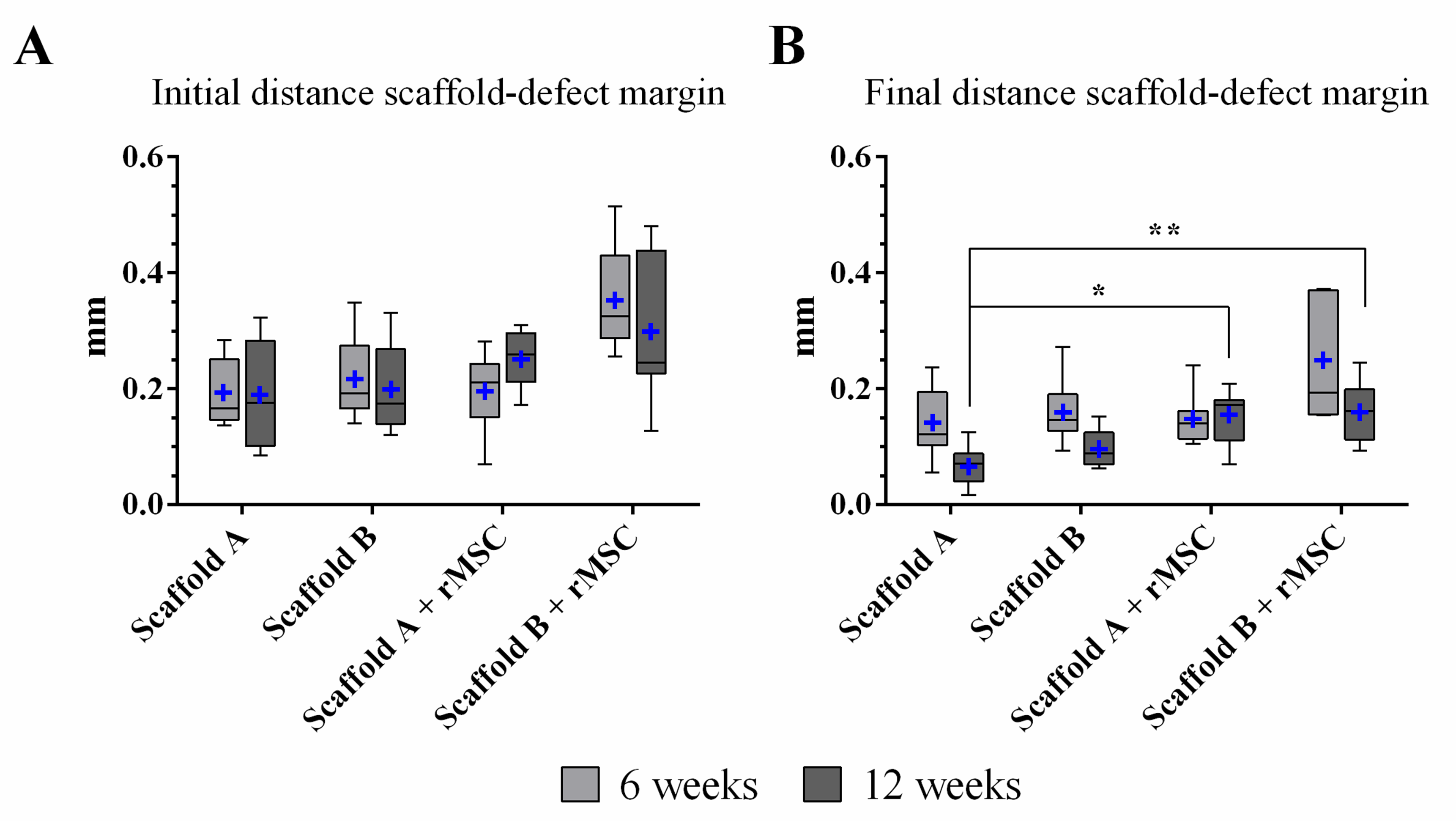

Supplement: FIGURE S1 — Histomorphometric analysis. Closest distance between scaffold and defect margin. (A) Initial distance at time point of implant placement. (B) Final distance at the end of the study after 6 or 12 weeks (median and minimum/maximum values, mean is marked by +, *p < 0.05, **p < 0.01). [file Image_1.TIF]
