# Supplementary material for: 3D Printing of Bone Grafts for Cleft Alveolar Osteoplasty – In vivo Evaluation in a Preclinical Model
Source: Front Bioeng Biotechnol. 2020 Mar 25;8:217. doi: 10.3389/fbioe.2020.00217 (PMC7109264; doi:10.3389/fbioe.2020.00217)
Supplement: TABLE S1 — Measurements in the histomorphological images (mean ± standard deviation) for the remaining defect width, bone formation and percentage of the newly formed bone related to the initial defect area after 6 and 12 weeks. [file Table_1.docx]

**Supplementary Material**

Bone formation in vivo

**Supplementary Table S1** lists the measured average values of the remaining defect width, bone formation and percentage of newly formed bone after 6 and 12 weeks, respectively. Furthermore, the closest distance between ingrowing bone and the scaffolds were measured to investigate whether bone gets formed constantly with ongoing healing time. The initial and final defect margin could be identified due to histomorphological criteria. The initial distance between scaffold and osseous defect margin measured minimal 0.115 mm (scaffold A) and maximal 0.227 mm (Scaffold B + rMSC) in the groups of 6 weeks healing time (**Supplementary Figure 1 A**). The values for the 12 week-groups ranked between 0.118 mm (Scaffold A) and 0.219 mm (Scaffold B + rMSC). There were no statistical differences at both time points. After 6 weeks, scaffold A exposed the smallest distance between osseous defect and scaffold margin (0.082 mm) and the widest gap could be observed for scaffold B + rMSC (0.135 mm), but the differences were not statistically significant (**Supplementary Figure 1 B**). With ongoing healing time, the distance decreased due to bone formation in all experimental groups (**Supplementary Figure 1 B**). Again, scaffold A showed the smallest values (0.027 mm), which was statistically significant compared to scaffold B + RMSC (0.122 mm; *p=0.002*) and scaffold A + rMSC (0.098 mm; *p=0.022*).

Supplementary Table S1. Measurements in the histomorphological images (mean±standard deviation) for the remaining defect width, bone formation and percentage of the newly formed bone related to the initial defect area after 6 and 12 weeks.

| **Group** | **N** | **Remaining defect width / [mm]** | | **Bone formation / [mm^2^]** | | **Percentage of the newly formed bone / [%]** | |
| --- | --- | --- | --- | --- | --- | --- | --- |
|  |  | 6 w | 12 w | 6 w | 12 w | 6 w | 12 w |
| **Control  (empty defect)** | 16 | 2.73±0.15 | 2.27±0.14 | 0.2±0.04 | 0.36±0.03 | 13.1±1.9 | 22.5±1.8 |
| **Scaffold A** | 16 | 2.77±0.14 | 2.28±0.14 | 0.17±0.04 | 0.35±0.04 | 8.2±1.8 | 19.0±1.8 |
| **Scaffold A + rMSC** | 16 | 2.91±0.14 | 2.75±0.14 | 0.18±0.03 | 0.21±0.04 | 7.7±1.8 | 8.7±1.8 |
| **Scaffold B** | 14 | 2.65±0.14 | 2.75±0.15 | 0.14±0.04 | 0.2±0.04 | 7.8±1.8 | 10.2±2.0 |
| **Scaffold B + rMSC** | 16 | 2.52±0.16 | 2.55±0.16 | 0.25±0.04 | 0.21±0.04 | 13.5±2.1 | 10.8±1.9 |

**Supplementary Table S2.** Measurements in the histomorphological images (mean±standard deviation) for the initial and final distance between bony defect margin and scaffold margin after 6 and 12 weeks.

| **Group** | **N** | **Initial distance scaffold-defect margin / [mm]** | | **Final distance scaffold-defect margin / [mm]** | |
| --- | --- | --- | --- | --- | --- |
|  |  | 6 w | 12 w | 6 w | 12 w |
| **Scaffold A** | 16 | 0.115±0.035 | 0.118±0.031 | 0.082±0.017 | 0.027±0.017 |
| **Scaffold A + rMSC** | 16 | 0.119±0.03 | 0.169±0.031 | 0.094±0.016 | 0.098±0.017 |
| **Scaffold B** | 14 | 0.148±0.031 | 0.216±0.033 | 0.017±0.135 | 0.079±0.018 |
| **Scaffold B + rMSC** | 16 | 0.227±0.035 | 0.219±0.032 | 0.135±0.019 | 0.122±0.087 |
